# A multiscale analysis of gene flow for the New England cottontail, an imperiled habitat specialist in a fragmented landscape

**DOI:** 10.1002/ece3.1068

**Published:** 2014-04-18

**Authors:** Lindsey E Fenderson, Adrienne I Kovach, John A Litvaitis, Kathleen M O'Brien, Kelly M Boland, Walter J Jakubas

**Affiliations:** 1 114 James Hall, Department of Natural Resources and the Environment, University of New HampshireDurham, New Hampshire, 03824; 2 United States Fish and Wildlife Service, Rachel Carson National Wildlife Refuge321 Port Road, Wells, Maine, 04090; 3 Maine Department of Inland Fisheries & Wildlife650 State Street, Bangor, Maine, 04401

**Keywords:** Connectivity, dispersal, early successional habitat, fragmentation, landscape genetics, New England cottontail, roads

## Abstract

Landscape features of anthropogenic or natural origin can influence organisms' dispersal patterns and the connectivity of populations. Understanding these relationships is of broad interest in ecology and evolutionary biology and provides key insights for habitat conservation planning at the landscape scale. This knowledge is germane to restoration efforts for the New England cottontail (*Sylvilagus transitionalis*), an early successional habitat specialist of conservation concern. We evaluated local population structure and measures of genetic diversity of a geographically isolated population of cottontails in the northeastern United States. We also conducted a multiscale landscape genetic analysis, in which we assessed genetic discontinuities relative to the landscape and developed several resistance models to test hypotheses about landscape features that promote or inhibit cottontail dispersal within and across the local populations. Bayesian clustering identified four genetically distinct populations, with very little migration among them, and additional substructure within one of those populations. These populations had private alleles, low genetic diversity, critically low effective population sizes (3.2–36.7), and evidence of recent genetic bottlenecks. Major highways and a river were found to limit cottontail dispersal and to separate populations. The habitat along roadsides, railroad beds, and utility corridors, on the other hand, was found to facilitate cottontail movement among patches. The relative importance of dispersal barriers and facilitators on gene flow varied among populations in relation to landscape composition, demonstrating the complexity and context dependency of factors influencing gene flow and highlighting the importance of replication and scale in landscape genetic studies. Our findings provide information for the design of restoration landscapes for the New England cottontail and also highlight the dual influence of roads, as both barriers and facilitators of dispersal for an early successional habitat specialist in a fragmented landscape.

## Introduction

Understanding how landscape features influence the connectivity and genetic variation of natural populations is of central importance in ecology, evolution, and conservation biology. Connectivity remains one of the most difficult parameters to measure, yet it is a critical issue to address in landscape conservation (Tischendorf and Fahrig 2000; Lindenmayer et al. 2008). From a species' perspective, connectivity is a function of the ability of an individual to disperse through the landscape. Characteristics of habitat patches and the intervening landscape matrix can either facilitate or impede dispersal success (e.g., Pérez-Espona et al. 2008). Because landscapes are spatially heterogeneous, and increasingly so as a result of human modifications, it is important to understand how landscape features affect animal movement and subsequent population processes.

Landscape influences on dispersal are determined by species-specific characteristics, including the organism's vagility and specific habitat requirements for dispersal. These factors determine the scale and extent to which specific landscape features influence population connectivity. For example, broadscale dispersal barriers may derive from natural landforms that are impassable, such as mountain ranges (Zalewski et al. 2009) or ocean trenches (Cunningham et al. 2009), and serve to completely separate populations. Local-scale or partial barriers are often formed by smaller landscape elements, such as roads (Coulon et al. 2006) or rivers (Frantz et al. 2010). The effects of these features can vary widely among species. Rivers may completely isolate populations of small mammals (Chambers and Garant 2010), but may be more permeable, at least under some circumstances, to larger mammals (Pérez-Espona et al. 2008; Cullingham et al. 2009) or even provide habitats conducive to dispersal (e.g., riparian corridors; Lowe and McPeek 2012). Similarly, roads, despite their recognized negative effects as barriers (Forman et al. 2003), may serve as movement corridors for some species for which associated habitat is conducive to dispersal (Crooks and Sanjayan 2006; Bissonette and Rosa 2009; Laurence et al. 2013). Linear landscape features may have complex influences on dispersal even within a single species, acting as both barriers and facilitators of dispersal. Anthropogenic changes in land cover can have further consequences for connectivity, as habitat loss and fragmentation can impede dispersal if the intervening matrix is prohibitive to a species' movement (e.g., Keyghobadi et al. 1999; Dixon et al. 2007). These consequences are more pronounced for species with high habitat specificity (Rothermel and Semlitsch 2002).

Disruption of habitat connectivity typically leads to genetic structuring among individuals, as a result of isolation (Segelbacher et al. 2003) and/or physical barriers to dispersal and concomitant gene flow (McRae et al. 2005). Reduced genetic exchange (i.e., fewer dispersing and subsequently reproducing individuals) among populations results in the gradual genetic divergence of populations through genetic drift and local adaptation (Willi et al. 2007) or, in the extreme, leads to population extinction (Bond et al. 2006). The consequences of reduced connectivity are especially relevant for species of conservation concern, which often exist in small, isolated patches and have limited dispersal and small effective population sizes (Ewers and Didham 2006). Small populations are more susceptible to stochastic events, as well as a loss of genetic diversity, which limits the population's ability to cope with environmental change (Templeton et al. 1990, 2001). In such cases, it is important to identify gene flow barriers that can be mitigated to increase effective dispersal. Improving connectivity helps maintain genetic diversity and increases effective population sizes, thereby strengthening the probability of population persistence (Newman and Pilson 1997; Frankham 2005; Bailey 2007). Additionally, recognizing landscape features that facilitate dispersal is necessary for species' recovery, so that those features can be maintained and replicated in habitat restoration efforts to increase connectivity and augment gene flow where needed.

Issues of connectivity are germane for organisms that rely on early successional and shrubland habitats. These ephemeral habitats occur in a landscape mosaic of habitats in varying successional stages, many of which are inhospitable to early successional habitat specialists. Although patchy by nature, the spatial configuration (abundance, patch size, and distribution) of early successional habitats has been modified by a loss of natural disturbance regimes, land use change, and anthropogenic landscape modifications. These habitats are on the decline in the northeastern United States, along with many species that rely on them (Brooks 2003; Litvaitis 2003; Lorimer and White 2003; Sauer et al. 2011). Consequently, early successional habitat specialists may face the consequences of habitat loss and fragmentation, including population isolation and decline, and concomitant reduction in genetic variation (Andren 1994; Fahrig 2003; Keyghobadi 2007).

**Figure 1:**
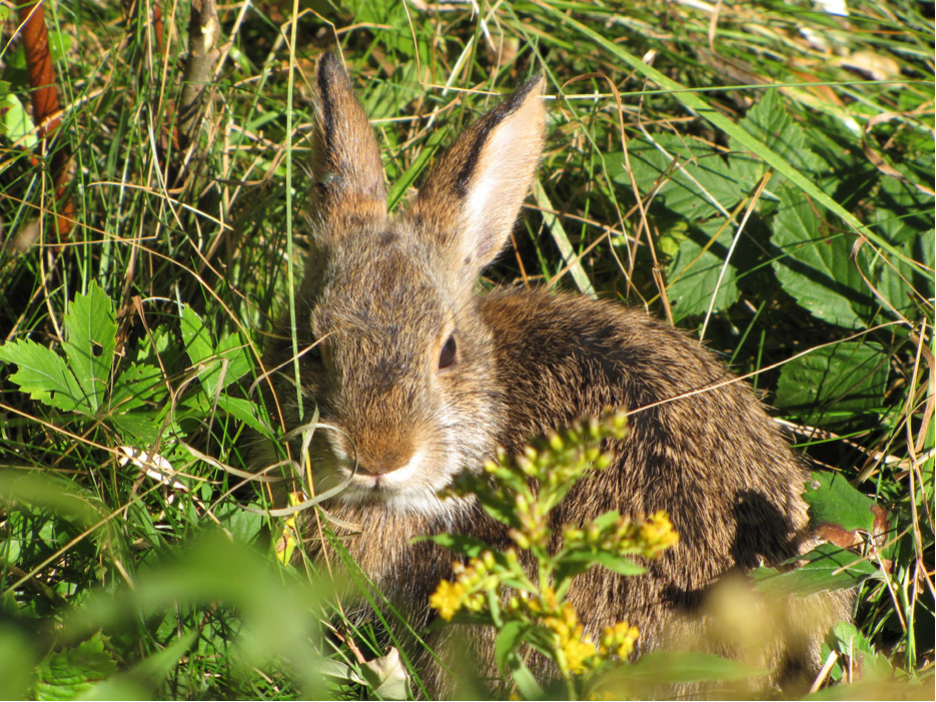
Young New England cottontail in old-field habitat. USFWS Photograph by Kelly Boland.

One of the many shrubland obligate species of high conservation priority in the northeastern United States is the New England cottontail (*Sylvilagus transitionalis*; Fig. 1), which requires dense, brushy vegetation for food and escape cover (Litvaitis et al. 2003). Widespread habitat loss has resulted in rapid population decline for this species, and it now occupies less than 14% of its historical range (all New England states and eastern New York; Litvaitis et al. 2006). As a result, the New England cottontail is listed as endangered in Maine (MDIFW 2007) and New Hampshire (NHFG 2008), and it is a candidate for federal listing under the Endangered Species Act (USFWS 2006, 2012). Remnant populations of New England cottontail currently occur in five geographically (Litvaitis et al. 2006) and genetically (Fenderson et al. 2011) isolated regions: (1) southern Maine and Seacoast (southeastern) New Hampshire; (2) central New Hampshire; (3) Cape Cod, Massachusetts; (4) eastern Connecticut and Rhode Island; and (5) western Connecticut, western Massachusetts, and eastern New York. The current population structure is a result of recent habitat fragmentation (within the last several decades) and genetic stochasticity, as the populations have experienced genetic drift in isolation (Fenderson et al. 2011).

Given the lack of gene flow among the remaining populations of New England cottontails, conservation efforts must begin within each of these regions to ensure connectivity, stability, and population persistence on a local scale. New England cottontails in southern Maine and the Seacoast region of New Hampshire are in immediate need of restoration management. This region is at the northern extent of the species' range and is experiencing ongoing decline, with an estimated 50% reduction in effective population size occurring within the past two decades (Fenderson 2010) and reduced genetic diversity relative to other remnant populations (Fenderson et al. 2011). A census population size of roughly 300 individuals has been estimated to occur in southern Maine (Litvaitis and Jakubas 2004), and an effective population size of 75–150 has been estimated for the Maine and New Hampshire region (Fenderson et al. 2011). Extensive habitat loss and fragmentation have reduced the availability of suitable (thicket) habitat in this region, such that fewer and smaller habitat patches exist, separated by increasingly large geographic distances. Remaining habitat patches are typically small (2–35 ha, with most <5 ha) and fragmented by development and inhospitable habitat. Recovery of the New England cottontail in the Maine and Seacoast New Hampshire region will require increasing available suitable habitat to support patch occupancy, as well as increasing connectivity among remaining patches. These efforts require an understanding of current landscape influences on gene flow.

The objectives of our study were threefold: (1) to assess local population genetic structure and diversity of New England cottontails in southern Maine and coastal New Hampshire; (2) to identify landscape features that are influential in structuring populations through promoting or inhibiting connectivity within and among these populations; and (3) to test hypotheses about the influence of landscape features (identified in #2) on gene flow. Specifically, we evaluated the effects of geographic distance, roads, waterbodies, and linear features comprised of early successional habitat, such as utility lines and roadsides, on gene flow. We expected to find fine-scale population structure resulting from the separation of populations by fragmentation and/or dispersal barriers. We hypothesized that landscape features have a stronger influence on genetic variation within and among populations than geographic distance alone. We also predicted that roads and waterbodies would function as dispersal barriers, while linear shrubby habitat features (railroads, powerline rights-of-way, and road*sides*) would facilitate gene flow. Our results provide key information for the design of restoration landscapes that enhance connectivity for the New England cottontail and thereby likely also benefit other species that rely on early successional habitats. Our findings also illustrate the complexity of natural and anthropogenic factors influencing gene flow of a habitat specialist in a fragmented landscape.

## Methods

### Study design and sample collection

We conducted systematic fecal pellet surveys across the recently occupied range of New England cottontails in southern Maine and Seacoast New Hampshire during the winters of 2007/2008 and 2008/2009. Surveying in the winter increases detectability due to the presence of tracks in the snow and the increased visibility of pellets (Brubaker et al. 2014). Cold temperatures promote preservation and yield of DNA in fecal pellets (Kovach et al. 2003). Additionally, winter sampling occurs after juveniles from the previous summer have dispersed (Chapman and Ceballos 1990) and prior to parturition of the first litter of the year (Chapman 1975). Sampling is thereby limited to postdispersal adults, and inadvertent sampling of highly related litter groups is avoided. The sampling of kin groups is further precluded by the solitary nature of New England cottontails (Litvaitis et al. 2008).

Sampling scheme and scale are important considerations in planning a landscape genetics study, and they can influence the conclusions reached (Anderson et al. 2010; Segelbacher et al. 2010). An ideal sampling scheme should incorporate the range of spatial and genetic variability by sampling a relatively fine grain size (with respect to an organism's dispersal distance) across a relatively large geographic area (Storfer et al. 2007; Schwartz and McKelvey 2009). For our objective of identifying landscape influences on a fine scale, a continuously distributed sampling scheme in areas of occupancy was appropriate (Storfer et al. 2007; Schwartz and McKelvey 2009). We surveyed 191 patches in 2007–2008 to determine occupancy and used these occupancy results as pilot data to plan sampling in the subsequent field season (Fenderson 2010).

**Figure 2:**
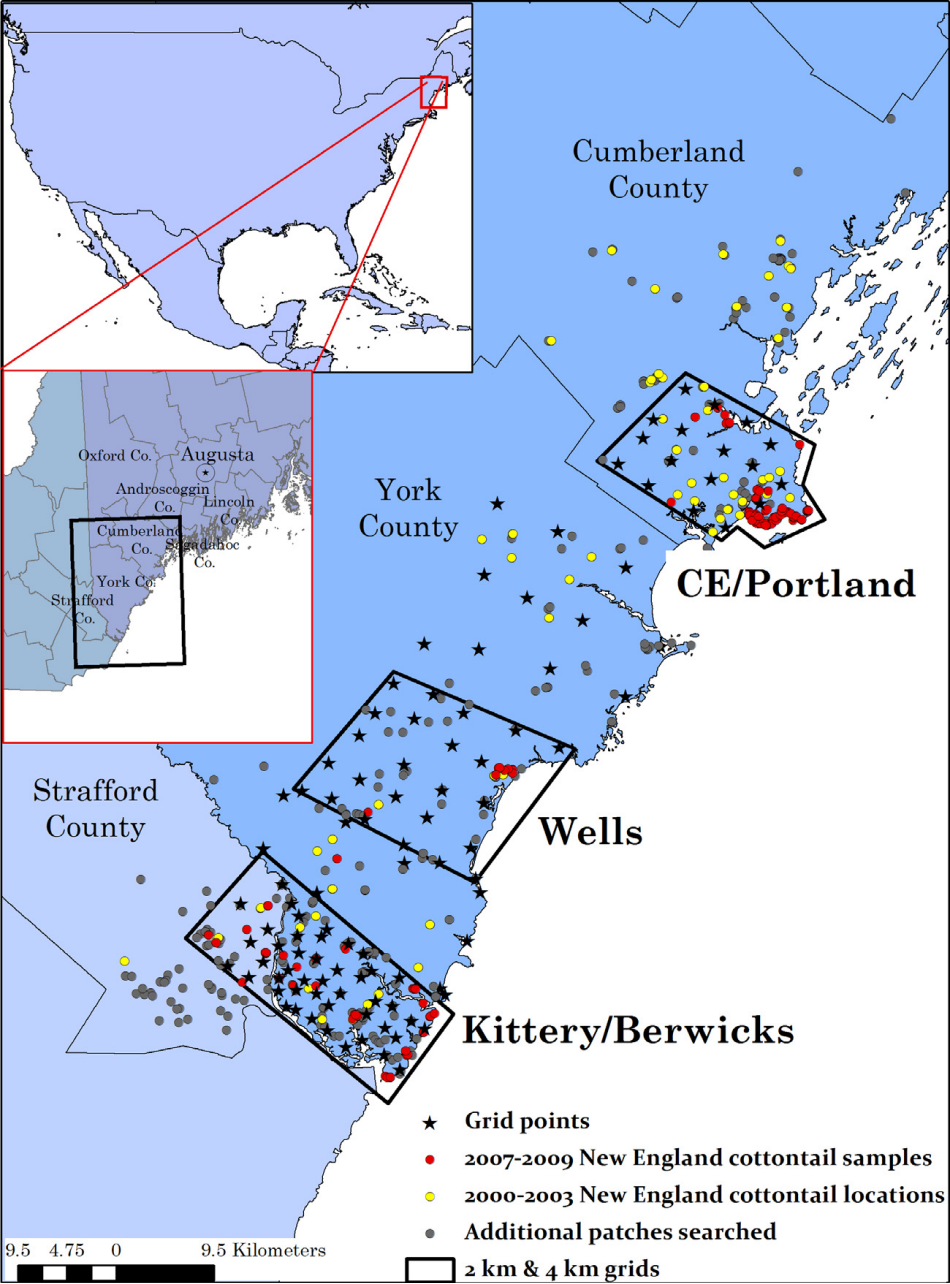
Sampling scheme for surveys of New England cottontail fecal pellets during the winter of 2008/2009 and all patches searched during both field seasons (2007/2008 and 2008/2009). Stars show the grid points used to center surveys (see text for explanation). Circles indicate all patches that were surveyed for this study. Red circles identify New England cottontail samples collected and yellow circles identify patches that were occupied by New England cottontails in 2000–2003, but no longer occupied in 2007/2008 or 2008/2009. Gray circles depict all of the remaining patches that were searched but were not occupied by cottontails.

Our sampling design in 2008–2009 was intended to obtain representative genotypes distributed continuously across the occupied landscape using a hierarchical systematic grid pattern. To optimize search effort and the number of unique individuals sampled, sampling was conducted using finer grains (1–2 km) in areas of recent occurrence and coarser grains (4–8 km) as the likelihood of encountering a New England cottontail decreased. Surveys were centered around grid points where we searched up to three suitable (densely shrubby) habitat patches within an approximate 1 km radius around each grid point if possible, although not all grid points had nearby suitable habitat (Fenderson 2010; Fig. 2). Within each occupied patch, we collected samples consisting of up to 10 pellets from a single pile or set of tracks, assumed to be from a single individual. Where possible, multiple samples were collected per patch, separated by at least 50 m, to maximize the number of individuals sampled. This was the most exhaustive sampling effort in this area to date and likely documented nearly all currently occupied New England cottontail patches in Maine and Seacoast New Hampshire. All pellets were stored at −20°C until analyzed. Also included in our dataset were three pellet samples collected in the winter of 2006/2007, seven opportunistically collected predator-kill or road-kill tissue samples, and blood samples from 19 animals trapped for relocation in 2010.

### Microsatellite genotyping

DNA was extracted from one pellet per sample using the QIAamp® DNA Stool Mini Kit (Qiagen, Valencia, CA), with minor modifications of the manufacturer's instructions as described in Kovach et al. (2003). As New England cottontails are sympatric with the eastern cottontail (*Sylvilagus floridanus*) and the snowshoe hare (*Lepus americanus*) in portions of their range, the species of origin of the pellets was determined using a combination of two diagnostic RFLP tests of the mitochondrial DNA, using the restriction enzymes *BfaI* (Litvaitis and Litvaitis 1996) and *NlaIII* (Kovach et al. 2003), following Fenderson et al. (2011) and Kilpatrick et al. (2013).

New England cottontail samples were amplified with fluorescent dye-labeled primers and multiplex protocols at 11 microsatellite loci (Table 1) in a two-tiered approach. First, we used eight loci previously found to be polymorphic in cottontails in this study area, including a SRY microsatellite for sex determination (Fenderson et al. 2011), to screen unique individuals from replicate samples collected within a patch. These eight-locus genotypes were compared in Dropout (McKelvey and Schwartz 2005) to identify unique individuals (*P*_*IDSIBS*_ for the seven autosomal loci = 1.711e-2; *P*_*IDSIBS*_ including the SRY locus = 1.135e-2). Samples from unique individuals were then genotyped at three additional loci determined to be polymorphic in this study (*Sfl8*, *Sfl11*, and *Sfl15;* Berkman et al. 2009). PCR products were electrophoresed on an ABI 3130 automated DNA sequencer (Applied Biosystems, Foster City, CA). Genotypes were manually scored using Peak Scanner 1.0 software (Applied Biosystems), and alleles were binned in reference to a positive control with the program Allelogram 2.2 (available at http://code.google.com/p/allelogram/), to ensure consistency of allele calls across multiple electrophoretic runs.

**Table 1:** Multiplex PCR conditions for microsatellite loci used in this study

Locus/Multiplex	Individual Primer Conc.	MgCl_2_	dNTP Conc.	Annealing Temp. (°C)	Cycles
Sat12/Lsa1	0.20 *μ*m	3.00 mmol/L	0.20 mmol/L	55	35
Sat13	0.33 *μ*m	1.50 mmol/L	0.20 mmol/L	55	38
Sat3	0.33 *μ*m	2.50 mmol/L	0.20 mmol/L	58	40
INRA016/Y	0.53 *μ*m	1.50 mmol/L	0.25 mmol/L	60	35
Sfl8^1^	0.40 *μ*m	1.00 mmol/L	0.20 mmol/L	58	30
Sfl11/Sfl15^1^	0.24 *μ*m	1.00 mmol/L	0.20 mmol/L	58	40
Sol44^1^	0.20 *μ*m	2.50 mmol/L	0.20 mmol/L	62	40
Sol03^1^	0.20 *μ*m	3.00 mmol/L	0.20 mmol/L	52	40

All PCRs were in 15 *μ*L reactions, except as noted. All reactions used 1X BSA, 1X buffer, and 0.75 U Taq polymerase.

1 12.5 *μ*L reaction.

To address issues of genotyping error, PCR amplification and electrophoresis were replicated at least three times for each sample at the eight initial loci and the three additional loci for unique samples, until a consensus genotype was reached. We required alleles to amplify at least twice for an individual to be scored as a heterozygote at a locus. Following Frantz et al. (2003), if this rule was not met with the initial three replicate PCRs, we repeated amplifications in a stepwise fashion, for up to seven replicates, until each allele was observed at least twice. If the DNA sample was exhausted before all replicate genotypes could be obtained, we still retained a genotype at a locus if it successfully amplified twice and an identical genotype was obtained each time (only 5% of the 8-locus consensus genotypes and 5% of the final 11-locus dataset were based on two amplifications). Samples missing data at four or more loci were excluded from analyses. To ensure unique individual identity of genotypes, we reinspected the raw genotype peaks of all pairwise samples that differed by ≤3 loci. Genotyping error was assessed by manually comparing each replicate genotype to the consensus (Taberlet et al. 1996) and calculating total error rates following Pompanon et al. (2005). We used Microchecker (Van Oosterhout et al. 2004) to test for null alleles with the Brookfield 1 estimator (Brookfield 1996) and Genepop (Raymond and Rousset 1995) to test for Hardy–Weinberg equilibrium (HWE) and gametic disequilibrium.

#### Population genetic structure, diversity, and effective size

We assessed population genetic structure using two Bayesian clustering methods: Structure (Pritchard et al. 2000), a program that delineates genetically similar individuals based solely on the genetic data and not on a priori population definitions, and Tess (Chen et al. 2007), a similar program that also incorporates sampling locations to help define genetic units. Structure was run 20 times at each *K* (the number of putative genetic populations) from 1–7 using a burn-in of 100,000 and run length of 500,000, with the no admixture and independent allele frequencies model (Pritchard et al. 2000). Tess was also run 20 times at each *K* from 2–7 for 600,000 iterations, including a burn-in period of 100,000 sweeps. We used the no admixture model and a spatial interaction parameter of 0.6 (Durand et al. 2009). For each analysis, the optimal *K* was determined from the plateau of the average lnPR(*X*|*K*) (Structure) or the average deviance information criterion (DIC) of each *K* plotted against *K* (Tess), and from evaluation of the bar plots. Individual population memberships were averaged using Clumpp 1.1.2 (Jakobsson and Rosenberg 2007) and visualized with Distruct 1.1 (Rosenberg 2004). Based on these results and geographic proximity, individuals were grouped into genetic clusters for the remaining analyses.

Gene flow among populations was assessed using assignment tests performed in Structure, using the genetic clusters as prior population information and the same burn-in and run length as above. Migration rate was set at 0.05, and we tested for migrant ancestry up to two generations back. Assignment tests were also performed in Geneclass 2.0 (Piry et al. 2004), with the Rannala and Mountain (1997) Bayesian method and a threshold of 0.05. We also conducted migrant detection using the L_home/L_max criterion with Monte Carlo resampling (Paetkau et al. 2004) and a threshold of 0.01. We estimated genetic differentiation among all pairs of population clusters using *F*_*ST*_, as implemented in FSTAT (Goudet 1995). False discovery rate (FDR) control (Benjamini and Hochberg 2000) was applied to assess significance for multiple comparisons using the Excel spreadsheet Tabulator (Verhoeven et al. 2005).

To evaluate genetic diversity within each population cluster, we used Genepop to calculate the number of private alleles in each population. Heterozygosities, allelic richness, and *F*_*IS*_ were calculated in Fstat, and private allelic richness was calculated in hp-rare (Kalinowski 2005). For multiple comparisons, we implemented FDR control, as above. Effective population size (*N*_*e*_) of each genetic cluster was calculated with two single sample methods: a linkage disequilibrium method performed in ldne (Waples 2006) and a Bayesian method performed in onesamp (Tallmon et al. 2008). We also tested for evidence of a genetic bottleneck in each of the clusters using several approaches. We used the M-ratio method with a Θ of 1, assuming a historical effective population size of 500, and the average parameter values identified by Garza and Williamson (2001) of a mean step size of 2.8 and the percentage mutations larger than single step of 0.12. We also utilized the one-tailed Wilcoxon signed-rank test for heterozygosity excess and tested for allelic mode-shift (Luikart et al. 1998) with Bottleneck (Piry et al. 1999). We set the variance to 12 and the percentage of single stepwise mutations to 0.88 (for consistency with the parameters used for M-ratio) and ran 1000 iterations.

#### Landscape influence on gene flow

Results of the Bayesian clustering analyses and additional preliminary analyses of Fenderson (2010) provided some insight into landscape features that may be influencing cottontail gene flow. Based on these results, we hypothesized that roads and waterbodies were limiting dispersal. Additionally, we hypothesized that several linear landscape features, including powerline rights-of-way, railroad edges, and roadsides, were conducive to dispersal because they tend to be comprised of shrub habitat (Tash and Litvaitis 2007). We tested our hypotheses more explicitly by developing several raster cost-distance models, to calculate pairwise individual effective geographic distances using the Cost Distance tool from the Landscape Genetics Toolbox in ArcGIS 10 (Etherington 2011).

We developed models for three feature classes: (1) A roads model tested the hypothesized barrier effects of roads by assigning them *elevated* costs relative to the background landscape matrix (all of the nonfeature cells that were assumed to have equal influence on cottontail dispersal). The variables of interest were the six classes of road, defined by traffic volume, and hypothesized to have increasing barrier effect with increasing traffic volume. (2) The surficial water model similarly evaluated the dispersal-limiting effects of waterbodies (including rivers, streams, lakes, ponds, and coastal inlets). Variables included the six Strahler stream order classes, hypothesized to have increasing barrier effect with increasing stream width; all other waterbodies without stream order information were grouped with the lowest stream order category. (3) The facilitators model assessed the effects of linear strips of shrubby habitat as facilitators of dispersal, by assigning these features *reduced* costs relative to the background matrix. Facilitator variable costs were based on expert opinion (i.e., biologists familiar with the study organism and its habitat preferences, based on extensive field experience), which considered railroads, class 3–6 road edges, class 1–2 road edges, and powerlines, in increasing order of hypothesized facilitating effect. In addition, we tested a null model of straight Euclidean distance. GIS data sources and additional details of methodology can be found in Appendix 1.

For each of the three feature classes (roads, surficial water, and facilitators), we developed four types of cost models – binary, linear, exponential, and logarithmic – to evaluate the relative dispersal cost of the variables in each feature class. In the binary model, all feature variable costs were equal, but higher (for the roads and surficial water barrier models) or lower (for the facilitator model) than the background cost. In the linear, exponential, and logarithmic models, the feature variable costs were ranked according to their hypothesized influence on dispersal (described above). Costs varied relative to one another in a linear, exponential, and logarithmic relationship, respectively, with increasingly higher costs given in the two types of barrier models and reduced costs in facilitator models on the variables hypothesized to have greater influence on dispersal (see Table 2 for model costs). Analyses were conducted for the study area as a whole as well as within each of the genetically distinct populations, as defined by the Bayesian clustering analyses. For the within-population analyses, we excluded the Jetport (see clustering results below), due to the small number of individuals sampled from a small geographic area with few intervening landscape features of interest.

**Table 2:** Costs used for feature variables in raster cost-distance analysis of 12 landscape models

Models	Binary	Linear	Exponential	Logarithmic
Roads^1^				
Background	1	1	1	1
Trail (Road Class 6)	10	2	4	10
Unimproved (Road Class 5)	10	3	9	100
Improved (Road Class 4)	10	4	16	1000
Secondary (Road Class 3)	10	5	25	10000
Primary (Road Class 2)	10	6	36	100000
Interstate (Road Class 1)	10	7	49	1000000
Surficial water^2^				
Background	1	1	1	1
Stream Order 1	10	2	4	10
Stream Order 2	10	3	9	100
Stream Order 3	10	4	16	1000
Stream Order 4	10	5	25	10000
Stream Order 5	10	6	36	100000
Stream Order 6	10	7	49	1000000
Facilitators^3^				
Powerlines	1	1	1	1
Road edges (Classes 1–2)	1	2	4	10
Road edges (Classes 3–6)	1	3	9	100
Railroads	1	4	16	1000
Background	10	5	25	10000

1 Relative costs were assigned according to the road classes.

2 Strahler stream order class was joined to National Hydrography Dataset waterbody and area files based on spatial location to take into account drainage as well as the size of the waterbody. This was used to assign relative costs, and all waterbodies without stream order information were assigned to the lowest stream order category (e.g., assigned a cost of “2” in the linear model).

3 Each facilitator variable was ranked by expert opinion according to its presumed utility in facilitating cottontail dispersal.

To evaluate the relationship between gene flow and the three landscape feature classes, we first used Mantel tests (Mantel 1967) to correlate the resulting cost distances with pairwise individual Rousset's *a*_*r*_ (Rousset 2000) genetic distances calculated in Spagedi (Hardy and Vekemans 2002). We log_10_-transformed the pairwise Euclidean distances and used the effective geographic distances untransformed. This analysis was conducted with the ecodist package (Goslee and Urban 2007) in R statistical software (R Development Core Team 2006). We ran 10,000 permutations using the nonparametric Spearman correlation. Significance was assessed following Bonferroni correction for multiple comparisons (adjusted *P*-value = 0.0038 for *α* = 0.05). We compared the Mantel *r* values for the four cost models for each of the three feature classes to evaluate which cost model exhibited the best linear relationship with genetic distance within each population.

Although Mantel tests are an appropriate method for comparing data consisting of distance measures (Legendre and Fortin 2010), their use in landscape genetic applications has come under recent scrutiny (Balkenhol et al. 2009; Guillot and Rousset 2013; Graves et al. 2013). Therefore, we used another complementary approach – multiple regression on distance matrices (MRM; Manly 1986; Lichstein 2007) – to evaluate the relative importance of the three landscape feature classes (roads, surficial water, and facilitators) and Euclidean distance in influencing New England cottontail gene flow. MRM, like Mantel tests, can be used with nonindependent, pairwise genetic distance data. However, it provides the advantage of examining the influence of all input matrices simultaneously and determining the statistical significance and relative importance of each variable of interest (Lichstein 2007). Balkenhol et al. (2009) found that, in simulations, MRM performed better than Mantel tests, with a good balance between type I error and power. MRM tests were conducted using the cost model (binary, linear, exponential, and logarithmic) with the highest Mantel *r* for each feature class, using 10,000 permutations and the Spearman correlation with the ecodist package in R. The relative influence of each feature class on genetic variation was further elucidated using the hierarchical partitioning method of Chevan and Sutherland (1991). This test was conducted in the hier.part package for R (Walsh and MacNally 2008) using the *R*^2^ values generated in the MRM analyses.

## Results

**Figure 3:**
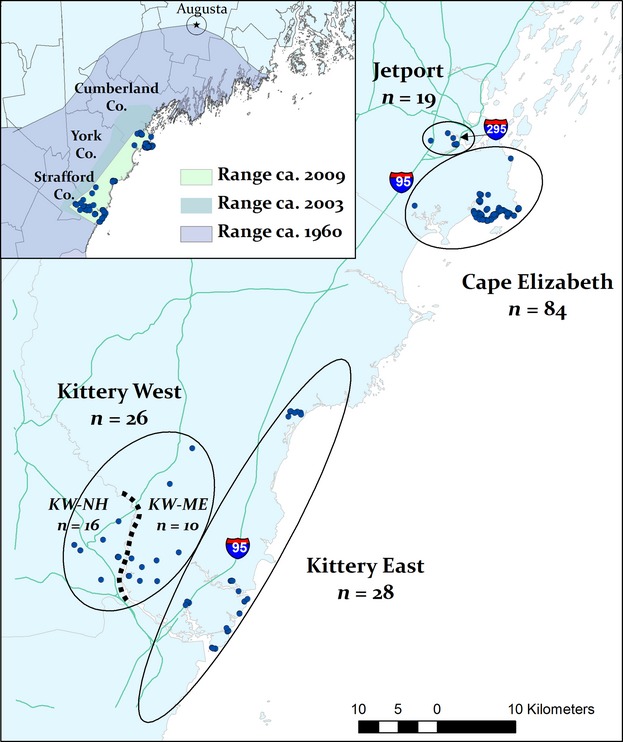
New England cottontail locations (points) and genetic clusters (circles) in southern Maine and New Hampshire. The dotted line indicates a partial barrier, consistent with the Salmon Falls/Piscataqua River, which further subdivides the Kittery West population into Kittery West-Maine (KW-ME) and Kittery West-New Hampshire (KW-NH). Inset: Estimated New England cottontail maximum range extents in this region circa 1960, 2003, and 2009 (this study), based on field surveys and historical reports.

Of 610 collected samples, 376 were identified as New England cottontail, and these samples originated from only 54 of 461 searched patches. These survey results revealed a significant range contraction in comparison with the most recent surveys of Litvaitis et al. (2006) conducted during 2000–2003 (Fig. 3). Of the 376 New England cottontail samples, 335 samples yielded sufficiently complete genotypes; 157 of those were determined to be unique individuals. Average raw genotyping error rates (our estimated genotyping error per single PCR replicate) across loci were 0.084 per genotype and 0.043 per allele (Table 3).

**Table 3:** Raw genotyping error rates^1^ (total allele scoring mismatches as compared to consensus genotype) for noninvasive New England cottontail fecal pellet samples at 10 autosomal and one Y-chromosomal microsatellite loci

Locus	Per-locus error rate	Per-allele error rate
Sat12	0.188	0.096
Sol03	0.161	0.083
Sol44	0.057	0.029
Lsa1	0.031	0.016
Sat13	0.151	0.078
Sat3	0.031	0.016
INRA016	0.070	0.035
Sfl11	0.035	0.018
Sfl15	0.046	0.023
Sfl8	0.023	0.012
INRA326 (Y)^2^	0.061	0.032

1 Error rates were calculated following Pompanon et al. (2005) equations 1 (e_a_ = m_a_/2nt; per-allele error rate) and 2 (e_l_ = m_l_/nt; per-locus error rate), where m represents the number of allelic (or genotypic) mismatches relative to the consensus genotype, *n* is the number of individual single-locus genotypes, and t is the number of replications.

2 Error rate for the SRY locus (INRA326) was based upon samples that produced an amplified product in more than one PCR run.

### Population genetic structure, diversity, and effective size

**Figure 4:**
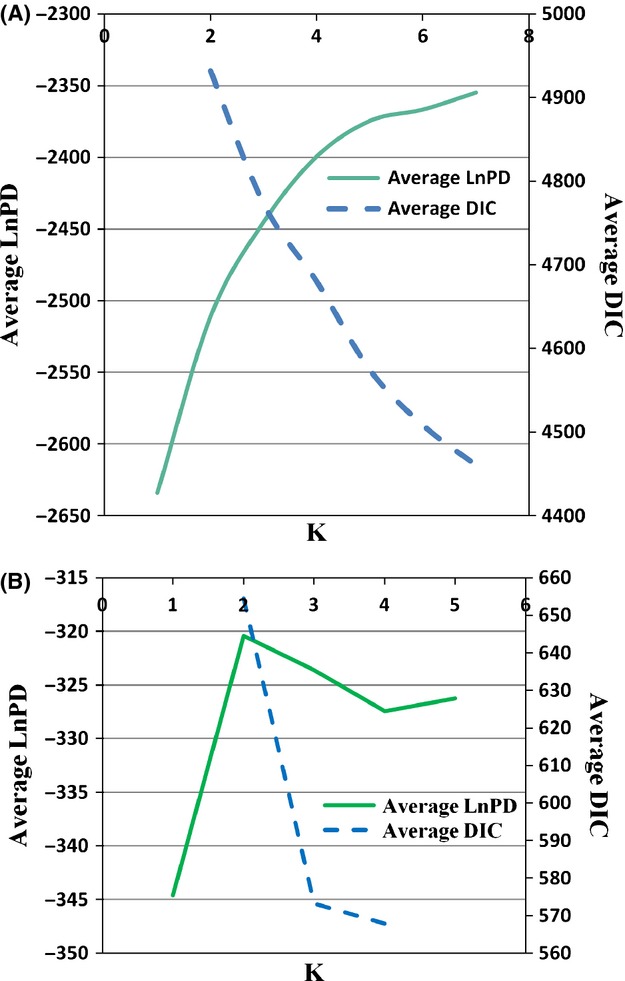
Determination of the number of New England cottontail genetic populations (*K*) in Maine and Seacoast New Hampshire based on Bayesian clustering results from Structure and Tess. For Structure, the number of putative populations is frequently determined by the highest average LnPD or where it begins to plateau. For Tess, the number of putative populations is also determined where the average deviance information criterion (DIC) begins to plateau and/or the *K* at which the Q-plots stabilize (Figure 5). Results are shown for the entire study area (A) and for Kittery West (B). See Results for our interpretation of the number of genetic clusters.

**Figure 5:**
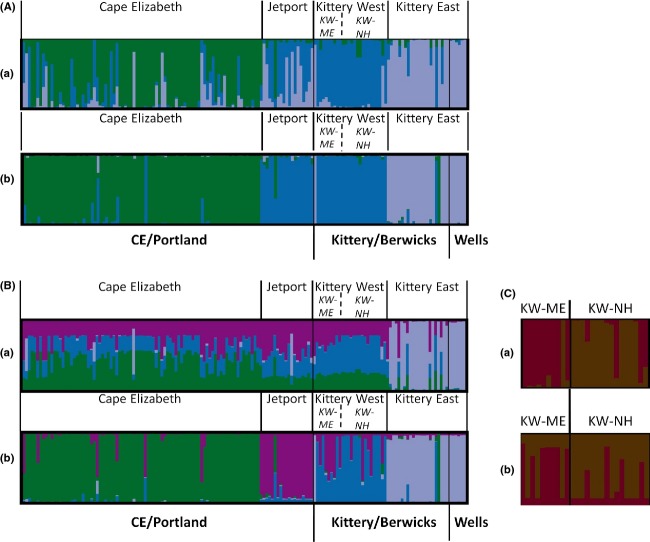
Individual assignment probabilities of New England cottontails to genetic clusters determined by a) Structure and b) Tess for (A) *K* = 3 and (B) *K* = 4. Geographic sampling locations are indicated below each pair of graphs in bold. Genetic cluster assignments are indicated above each graph. Subdivisions of the Kittery West population are shown in C).

The Bayesian clustering methods detected hierarchical population genetic structure (Figs 4 and 5). For *K* = 3, the bar graphs in both Structure and Tess identified support for three differentiated populations: (1) a large cluster of individuals from Cape Elizabeth (Cape Elizabeth; Fig. 3); (2) the individuals sampled at the Portland International Jetport (Jetport) together with those sampled on the western side of I-95, including Seacoast New Hampshire (Kittery West); and 3) all of the individuals sampled from east of I-95, as well as the individuals from a patch that directly abutted the interstate on the western side (Kittery East). Structure bar graphs seemed to best support *K* = 3 as above; however, the LnPD began to plateau at *K* = 5, suggesting the potential for finer-scale structure (Fig. 4). For Tess, at *K* = 4 the DIC values showed a slight plateau and the bar graphs stabilized, with the Jetport differentiated into a separate cluster from Kittery West, albeit with some mixed ancestry (Fig. 5). Tess hard-clustering results indicated a subdivision in Kittery West that seemed to approximate the geopolitical boundary between Maine and New Hampshire that is formed by the Salmon Falls and Piscataqua Rivers (data not shown). Further, the individuals with shared ancestry between the Jetport and Kittery West were sampled northeast of the river in Maine. Given the large geographic distance (48 km) between the Jetport and the closest patch of individuals in Kittery West today, we considered these findings to reflect a historical connection between these populations and determined it more biologically meaningful to view them as separate clusters with respect to current occupancy patterns. That is, despite evidence for their recent connectivity, cottontails in these populations are functionally separate populations today with genetic drift acting independently within each population. Lack of current migrants detected between these two populations (see assignment test results below) further supported this separation.

To test for finer-scale structure, we conducted an additional analysis with both Structure and Tess on just the Kittery West individuals, using the same parameters as before, up to *K* = 4. We found support for additional substructure within Kittery West, comprising two populations: the individuals sampled in Maine, northeast of the river (KW-ME), and those individuals sampled from Seacoast New Hampshire, west of the river (KW-NH; Fig. 3). Combining inference from the above analyses, we concluded there are four major genetic clusters of New England cottontails (Cape Elizabeth, Jetport, Kittery East, and Kittery West), with weaker substructure within Kittery West comprising two subpopulations (Fig. 3). Based on these results, individuals were grouped for the remaining analyses according to the dominant genetic cluster assignment of its sampling location. Downstream analyses were conducted both for *K* = 4 (considering Kittery West as one population) and for *K* = 5 (keeping separate the two subdivisions in Kittery West) where we deemed it relevant.

Microchecker analyses found three loci with null allele frequencies >10% in at least one population: *Sol03* in Cape Elizabeth (11.2%), *Sfl11* in Kittery East (14%), and *Sfl8* in Cape Elizabeth and Kittery West (17.3% and 18.6%, respectively). No null alleles were detected in KW-ME or KW-NH. *Sol03* and *Sfl8* were out of Hardy–Weinberg equilibrium in the Cape Elizabeth population; all remaining loci and populations exhibited HWE, lending support to the genetic cluster designations. As null alleles have minor impacts on genetic distance measures (Chapuis and Estoup 2007), we retained these loci for downstream analyses. In the Cape Elizabeth population, *Sol03* and *Sfl8* also exhibited gametic disequilibrium, likely due to the null alleles, while *Sat13/INRA16* and *Sat13/Sfl11* were not in equilibrium in the Kittery East population. The latter effect is likely due to the null alleles in *Sfl11* and a possible Wahlund effect in Kittery East, which includes a geographically isolated group of cottontails in the Wells area. Linkage disequilibrium is often found in small populations, especially as a result of recent isolation and subdivision (Frankham et al. 2002; Zartman et al. 2006).

Genetic diversity measures were similar for each of the genetic clusters (Table 4). Private alleles were identified in each cluster except KW-NH. *F*_*IS*_ values were significantly higher than zero for *Sol03*, *Sfl11*, and *Sfl8*, due to the null alleles, leading to significant *F*_*IS*_ in the Cape Elizabeth, Kittery East, and Kittery West populations overall. When calculated without the three null allele loci, *F*_*IS*_ was not significant for any population. All pairwise *F*_*ST*_ values were significant (overall *F*_*ST*_ = 0.127, *P* < 0.001), and the largest differences occurred in comparisons of KW-NH and the other populations (Table 5).

**Table 4:** Genetic diversity of New England cottontail loci and genetic clusters in southern Maine and New Hampshire. Alleles, allelic richness, observed heterozygosity (*Ho*), unbiased expected heterozygosity (*UHe*), and *F*_*IS*_ are across all samples (per-locus data) or averaged across loci (per-population data). Private alleles are the total number (and private allelic richness is the sample size adjusted proportion) of private alleles for all loci in each population

Locus	Alleles	*Ho*	*UHe*	*F*_IS_
Sat12	6	0.689	0.758	0.091
Sol03	6	0.571	0.750	0.238*
Sol44	4	0.588	0.582	−0.011
Sat13	6	0.575	0.598	0.098
Lsa1	3	0.201	0.223	0.038
Sat3	5	0.273	0.339	0.196
INRA016	2	0.276	0.248	−0.113
Sfl11	4	0.356	0.462	0.229*
Sfl15	2	0.410	0.484	0.154
Sfl8	2	0.159	0.385	0.589*

		Allelic richness				Private alleles		
						
Population (*N*)	Alleles	4 pops.	5 pops.	Ho	UHe	4 pops.	5 pops.	*F*_*IS*_^1^
Cape Elizabeth (84)	3.2	2.99	2.70	0.443	0.484	3 (0.25)	3 (0.24)	0.086*/−0.034
Jetport (19)	2.5	2.49	2.21	0.337	0.323	3 (0.29)	3 (0.15)	−0.044/−0.045
Kittery East (28)	3.1	2.99	2.68	0.384	0.433	1 (0.13)	1 (0.18)	0.115*/0.076
Kittery West (26)	3.0	2.85		0.379	0.424	2 (0.22)		0.110*/0.037
KW-ME (10)	2.6		2.52	0.441	0.462		1 (0.19)	0.047/−0.032
KW-NH (16)	2.8		2.35	0.340	0.334		0 (0)	−0.021/−0.021

* Indicates *P* < 0.05 after Bonferroni correction.

1 Population level *F*_*IS*_ values are given for both the full 10-locus dataset and without the three loci with null alleles (Sol03, Sfl11, and Sfl8), before and after the forward slash, respectively.

**Table 5:** Pairwise *F*_*ST*_ among New England cottontail genetic clusters in Maine and Seacoast New Hampshire

	Cape Elizabeth	Jetport	Kittery East	KW-ME
Cape Elizabeth				
Jetport	0.087			
Kittery East	0.112	0.102		
KW-ME	0.087	0.071	0.127	
KW-NH	0.165	0.231	0.244	0.171

All *F*_*ST*_ values were significant at the 5% level.

The two Kittery West subpopulations were separated for assignment tests and migrant detection. The Geneclass assignment test assigned 87.3% of the individuals back to their sampled location (quality index = 82.75%), and only six individuals were cross-assigned to other populations with relatively high probability (>75%). Only two individuals were identified as migrants by both Geneclass and Structure. One was an individual sampled in Kittery East that assigned to KW-NH, and the other was sampled in KW-ME and assigned to KW-NH. Five other individuals were assumed to have admixed ancestry based on meeting at least two of the following criteria: (1) >50% Geneclass assignment probability to a cluster other than that of geographic origin; (2) identified as a putative first-generation migrant with Geneclass migrant detection; (3) <50% Structure resident probability; and/or (4) >10% Structure immigrant probability. Three additional individuals from Cape Elizabeth were cross-assigned to the Jetport with >85% probability in Geneclass; however, they had >85% resident probability in Structure. Due to the proximity of the two populations, we also considered them as potentially admixed.

Effective population sizes for each cluster ranged from only 3.2 in the Jetport to 36.7 in Cape Elizabeth (sample sizes were too small to test the Kittery West subpopulations separately; Table 6). Estimates obtained by the two methods were significantly different for Kittery East (based on nonoverlapping 95% confidence intervals). The Cape Elizabeth population showed signs of having experienced a recent genetic bottleneck (Table 6). It exhibited significant heterozygosity excess by the Bottleneck method under the I.A.M. and T.P.M. mutation models, with the Wilcoxon one-tailed probability test. Kittery East and KW-ME also had significant heterozygosity excess under the I.A.M. model, and KW-ME had a shifted allelic mode distribution as well. The M-ratio method also detected a significant genetic bottleneck in KW-ME.

**Table 6:** Estimated effective population sizes (mean and 95% CI of *N*_*e*_) using the LDNe and ONeSAMP estimators and results of genetic bottleneck tests using Bottleneck and M-ratio methods for New England cottontail genetic clusters in southern Maine and Seacoast NH

Population	Effective population size estimates^1^		Bottleneck^2^ Wilcoxon Test Probability			Bottleneck Mode-Shift	M-ratio^3^	Mc (*N*_*e*_ = 500)	M-ratio Probability
	
	Mean *N*_*e*_ (95% CI) LDNe	Mean *N*_*e*_ (95% CI) ONeSAMP	IAM	TPM	SMM				
Cape Elizabeth	36.7 (22.0–67.9)	35.0 (24.0–71.9)	0.001*	0.012*	0.097	Normal L-shaped	0.865	0.815	0.21
Jetport	3.2 (1.6–13.9)	14.1 (11.1–18.6)	0.326	0.787	0.898	Normal L-shaped	0.829	0.798	0.13
Kittery East	5.3 (2.5–10.9)	28.3 (21.0–59.0)	0.016*	0.313	0.348	Normal L-shaped	0.828	0.802	0.11
Kittery West^4^	16.8 (6.1–123.7)	23.9 (17.2–50.0)	0.138	0.615	0.754	Normal L-shaped	0.814	0.802	0.08
KW-ME			0.042*	0.313	0.423	Shifted Mode	0.781	0.785	0.04*
KW-NH			0.754	0.947	0.958	Normal L-shaped	0.830	0.797	0.15

* *P* < 0.05.

1 Effective population sizes could not be estimated for the Kittery West subpopulations due to low sample size.

2 For the tests performed in Bottleneck, the Wilcoxon one-tailed probability of heterozygosity excess for three mutation models (IAM = infinite allele model; TPM = two-phase model; and SMM = stepwise mutation model) is given, as well as results of the allelic mode-shift test.

3 The M-ratio for each genetic cluster is specified; critical M values (Mc) were calculated using *N*_*e*_ = 500; the M-ratio probability is the probability that the M-ratio is significantly lower than the Mc value.

4 Bottleneck probabilities for Kittery West are from a separate simulation as the other five populations.

### Landscape influence on gene flow

**Figure 6:**
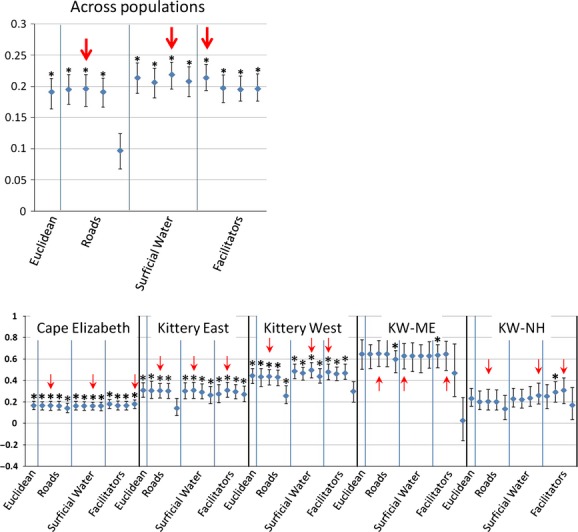
Mantel *r* correlations and 95% confidence intervals of genetic distance and effective geographic distance for cost models testing the influence of three types of landscape features on gene flow in New England cottontails. Roads and surficial water models tested hypothesized barrier effects of these features on dispersal, and the facilitator models tested the hypothesized influence of linear conduits in promoting dispersal. For each feature class, four cost models were tested (from left to right): binary, linear, exponential, and logarithmic, evaluating the relative cost of each feature variable on dispersal. For comparison, correlation with Euclidean distance alone (a model of isolation by distance) is shown. Statistical significance (indicated by asterisks) was assessed with 10,000 permutations and two-tailed *P* < 0.05 following Bonferroni correction (corrected *P* < 0.0038). Arrows indicate the model within each feature class with the highest Mantel *r*. Top panel – across the Maine-New Hampshire study area as a whole; bottom panel – within each genetically distinct population.

For the Mantel tests comparing our hypothesized cost distances with cottontail genetic distance across the entire study area, all of the dispersal models, except the logarithmic road model, were statistically significant. The significant Mantel correlations ranged from 0.1915 to 0.2159 and were slightly stronger than the correlation with Euclidian distance (*r*_*M*_ = 0.1913); however, all confidence intervals overlapped (Fig. 6).

Within the genetic clusters, the results of the Mantel tests were more varied and no single cost model performed the best for all feature classes (Fig. 6). For the roads feature class, the linear cost models performed the best in each population, while the logarithmic road models were always the least correlated with genetic distance. For the surficial water and facilitator models, each cost model performed best in at least one of the populations. In Cape Elizabeth, Kittery East, and Kittery West, all models were significant. In Cape Elizabeth, all facilitator models had stronger correlations with gene flow than did Euclidean distance, and in Kittery East, only the linear surficial water and linear facilitator models were slightly more correlated with cottontail genetic distance than was Euclidean distance. For Kittery West, except for the logarithmic models, all of the facilitator and surficial water models explained more variation in genetic distance than Euclidean distance alone. For the Kittery West subpopulations, very few models were significant, likely due to low sample size. Only the linear road model performed better than the Euclidean model in KW-ME, whereas in KW-NH, most of the facilitator models, as well as the exponential and logarithmic surficial water models, performed better than the Euclidean model, but only the linear facilitator model was significant.

**Figure 7:**
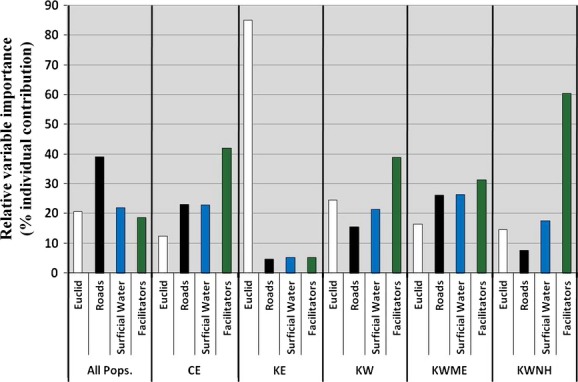
Hierarchical partitioning of the independent effects of Euclidean distance and three landscape feature class types on New England cottontail genetic distance across the study area and within each population in southern Maine and Seacoast New Hampshire.

The MRM analysis allowed for quantitation of relative importance of the features in each population. The full models (which included the cost model with the highest Mantel correlation for each of the three feature classes and the Euclidean distance model) were significant across all populations and within each population, except KW-NH, although they explained a small amount of the total genetic variation (0.3–10%). For the study area as a whole, only the road variable had a significant positive association with genetic distance, and Euclidean distance had a significant, but negative, association (Table 7). For the analyses within the genetic clusters, however, the road variables were not significant in any population. Facilitator features were highly significant for Cape Elizabeth and Kittery West, as well as for KW-NH and marginally so for KW-ME. Euclidean distance had a significant negative correlation in Kittery West and a significant positive correlation in Kittery East. Surficial water was also positively associated with genetic distance for Kittery West. Hierarchical partitioning of the independent effects of each of the features showed that, across the study area, nearly half of the explained variance in cottontail genetic distance was due to the influence of roads, and the independent effects of geographic distance, surficial water, and facilitating habitat were about equal (Fig. 7). Within the genetic clusters, facilitating features explained the greatest percentage of the genetic variation for all populations except Kittery East, which showed a strong influence of Euclidean distance alone.

**Table 7:** Multiple regression on matrices (MRM) analysis of the influence of three types of landscape features – roads (Rd), surficial water (Rvr), and facilitating habitat (Facil) – and Euclidean distance (Euclid) on New England cottontail gene flow across and within five populations in southern Maine and New Hampshire. The full models were constructed using the cost model – binary (Bnry), linear (Lnr), exponential (Exp), and logarithmic (Log) – with the highest Mantel correlation for each feature class. *R*^*2*^ values are given for each full model and *β* values for each variable in the model. Significant *P* values (*P* < 0.05) are indicated in bold

Genetic cluster	Model	MRM *β*	*β P*-value	*R*^2^	*R*^2^ *P*-value
All populations	Euclid + RdLnr + RvrExp + FacilBnry			0.0645	**0.0001**
	Euclid	−0.2999	**0.0145**		
	RdLnr	0.7429	**0.0007**		
	RvrExp	−0.0621	0.7651		
	FacilBnry	−0.1847	0.1634		
Cape Elizabeth	Euclid + RdLnr + RvrExp + FacilLog			0.0032	**0.0001**
	Euclid	−0.0085	0.5653		
	RdLnr	0.0355	0.2864		
	RvrExp	0.0036	0.9117		
	FacilLog	0.0353	**0.0001**		
Kittery East	Euclid + RdLnr + RvrLnr + FacilLnr			0.0293	**0.0001**
	Euclid	0.2457	**0.0001**		
	RdLnr	0.0073	0.5864		
	RvrLnr	−0.2073	0.1820		
	FacilLnr	−0.0004	0.9969		
Kittery West	Euclid + RdLnr + RvrExp + FacilBnry			0.0435	**0.0001**
	Euclid	−0.0918	**0.0251**		
	RdLnr	0.0762	0.0603		
	RvrExp	0.1041	**0.0001**		
	FacilBnry	0.1287	**0.0001**		
KW-ME	Euclid + RdLnr + RvrBnry + FacilLnr			0.1037	**0.0003**
	Euclid	−0.0076	0.9545		
	RdLnr	−0.2557	0.6325		
	RvrBnry	0.4061	0.4340		
	FacilLnr	0.1959	0.0537		
KW-NH	Euclid + RdLnr + RvrLog + FacilExp			0.0088	0.1354
	Euclid	0.0392	0.6196		
	RdLnr	0.0102	0.8940		
	RvrLog	−0.0756	0.2349		
	FacilExp	0.0862	**0.0237**		

## Discussion

Habitat loss and fragmentation can alter the genetic structure and diversity of natural populations through a disruption of gene flow and metapopulation processes (Gonzalez et al. 1998). Effects are most pronounced in species with strong habitat associations, for which fragmentation impedes dispersal (e.g., Rothermel and Semlitsch 2002). New England cottontail populations have been declining for decades as a result of ongoing loss and fragmentation of early successional habitats (Litvaitis et al. 2006, 2008). The results of this study suggest that reduced occupancy is associated with low genetic connectivity among fragmented populations of New England cottontails in Maine and Seacoast New Hampshire. The current distribution of New England cottontails in this region represents a substantial range contraction since previous surveys in 2000–2003, when cottontails were found as far north as Cumberland, Maine (20 km north of the current northernmost location in Cape Elizabeth, Maine), and also occupied patches in the intervening region between the three disjunct, currently occupied areas (Litvaitis et al. 2006; Figs 2 and 3). This range contraction, combined with our findings of genetically isolated populations with low genetic diversity, emphasizes the immediacy of restoration needs for New England cottontails in Maine and New Hampshire.

### Genetic structure, diversity, and bottlenecks

New England cottontails in Maine and Seacoast New Hampshire were structured into five genetically distinct and geographically separated populations, the boundaries of which coincided with major highways, urban development, and rivers. Although Bayesian clustering results indicate recent, historical connections, gene flow is currently absent or very minimal among these populations, as evidenced by assignment tests and the relatively strong differentiation (significant *F*_*ST*_ values) among all pairs of populations. The presence of private alleles in each population further suggests that rapid genetic drift is occurring in the absence of dispersal. The distances separating populations greatly exceed the estimated mean dispersal distances of New England cottontails (500 m–3 km; Litvaitis and Villafuerte 1996; Fenderson 2010) and even the maximal dispersal distances of other lagomorphs (12–17 km; Gillis and Krebs 1999; Estes-Zumpf and Rachlow 2009; Bray et al. 2007). While long-distance dispersal events are important in population dynamics of small mammals (Diffendorfer and Slade 2002), it is unlikely that cottontails can disperse the current interpopulation distances necessary to maintain gene flow among remnant populations.

Fragmentation and subsequent population isolation have had negative genetic consequences for New England cottontails in Maine and Seacoast New Hampshire. Fenderson et al. (2011) found that genetic diversity, as measured by allelic richness (*Ar* = 2.6–2.9) and heterozygosity (*H*_*o*_ = 0.223–0.287), was reduced in Maine and New Hampshire relative to geographic areas in the core of the species' range (*Ar* = 3.4–4.0 and *H*_*o*_ = 0.371–0.492 for eastern Connecticut/Rhode Island and western Connecticut/New York), while common ancestry estimates (*F*-values) were increased (0.19 in Maine/New Hampshire compared to 0.09–0.12). Genetic diversity was similarly low across the four populations identified in this study, with slightly reduced allelic richness and heterozygosity in the Jetport relative to the other populations. The reduced genetic diversity in the Jetport is consistent with the origin of these 19 individuals from a single habitat patch in an isolated area. Genetic diversity has been found to have important effects in determining population dynamics (Reed et al. 2007), warranting further investigations into the implications of low genetic diversity on individual fitness and potential inbreeding effects on cottontail populations. Concerns about inbreeding may be germane in light of our subsequent research findings of high genetic similarity among cottontails on some small patches in Maine and New Hampshire (Brubaker 2012; A. Kovach unpubl. data).

Further genetic consequences are evidenced by genetic bottleneck tests, which indicated that several of the populations have recently experienced a demographic bottleneck or possibly are currently undergoing one. Of the two methods we used, the Bottleneck approach is more sensitive for detecting recent bottlenecks (within a few dozen generations), while the M-ratio test is best for detecting more historic or longer-duration bottlenecks (Williamson-Nateson 2005). Our results are most consistent with recent bottleneck effects, with the exception of KW-ME, which showed significance with both Bottleneck methods and the M-ratio test. This might indicate that the bottleneck effects are most severe in this population, which is bounded on the west by the river and on the east by the interstate and is now effectively isolated from the northern population. The evidence for a recent bottleneck was also strong in Cape Elizabeth, the northernmost New England cottontail population rangewide, as even the highly conservative stepwise mutation model approached significance. The lack of significance with the M-ratio method in the Cape Elizabeth population supports a recent demographic decline, consistent with our documented range contraction in the last decade, its current separation from the nearest population to the south by 29 km, and its isolation from the north and west by interstates. Populations at the periphery of a species' geographic range often have reduced gene flow, genetic variation, and effective population sizes (Schwartz et al. 2003), which are often manifest in genetic signatures of bottlenecks.

Along with low genetic diversity and bottleneck signatures, we found evidence of low effective population sizes for cottontails in this region. Previously, Fenderson et al. (2011) estimated the effective population size for the entire Maine/New Hampshire population (including a small cluster of individuals in central New Hampshire) to be 75–150 individuals. With further analysis, we have found that cottontails in this region actually occur in several small populations with critically low effective population sizes of <40 individuals. The lower the effective population size, the greater the likelihood of negative genetic consequences, such as inbreeding and extinction through stochastic effects (Allendorf and Ryman 2002). Conventionally, an effective population size of at least 50 is suggested for short-term persistence, while an effective size of 500 is considered necessary to maintain long-term evolutionary potential (Franklin 1980; Franklin and Frankham 1998; Jamieson and Allendorf 2012). Lagomorphs, however, may require an effective size of >300 to even persist for 40 generations (based on a census size of 3000 – Newmark 1987; Reed et al. 2003; assuming a conservative *N*_*e*_*/N* ratio of 0.1, Frankham 1995). Within this context, New England cottontails in the Maine and Seacoast New Hampshire region currently do not exist in populations large enough to persist into the near future.

Lagomorphs as a taxon may be particularly vulnerable to extinction, likely due to their short generation times and large fluctuations in population size, despite their high growth rates (Newmark 1995). The survival advantages for species with high growth rates persist only at large population sizes, and high growth rate species have higher extinction risk than lower growth rate species at small population sizes (Pimm et al. 1988; Newmark 1995). Low population sizes may act synergistically with poor habitat quality, such as that resulting from anthropogenic influence, to further increase extinction vulnerability (Reed et al. 2003). Reduced genetic variation and effective population size may negatively impact survival, fecundity, and population growth rates (Reed et al. 2007). These circumstances are important to consider in predicting the future persistence of New England cottontails in Maine and Seacoast New Hampshire, where the limited, degraded, and fragmented suitable habitat, combined with reduced genetic diversity, likely exacerbates the vulnerability of these small populations.

### Anthropogenic and natural influences on gene flow

Extensive movements in a fragmented landscape likely come at significant costs in the form of increased energy expenditure and high mortality risks. Even distances >5 km may be difficult for cottontails to overcome, as we previously found genetic discontinuities associated with this level of patch isolation (Fenderson 2010). Successful dispersal among disjunct patches is likely strongly dependent on the intervening landscape matrix. In this study, we found that three matrix features – roads, waterbodies, and linear conduits of thicket habitat – influenced gene flow of cottontails. The relative importance of each feature type, however, was a function of the landscape matrix at the scale of analysis and varied by population, illustrating the effects of scale and landscape gradients (Schwartz and McKelvey 2009; Cushman and Landguth 2010; Jaquiery et al. 2011).

At all spatial scales, roadsides and other facilitating habitat features had positive effects, while roads, waterbodies, and geographic distance had negative effects on cottontail gene flow. Across the study area as a whole, major highways, the river, and geographic isolation subdivided cottontail populations, while within populations, features that facilitate dispersal between suitable habitat patches were important in maintaining gene flow on a local scale. In Cape Elizabeth, where occupied patches were large and proximate and the landscape matrix contained few dispersal-limiting features (few waterbodies and only low-volume roads), only facilitating habitat was important in explaining gene flow. In Kittery East, which is the most fragmented population, comprised of two disjunct clusters of individuals separated by 20 km and where remnant patches are small (average patch size is 2.1 ha compared to 3.9 ha in Kittery West and 5.4 ha in Cape Elizabeth; Fenderson 2010), geographic distance was the most important factor. In Kittery West, the dispersal-limiting effect of a large river dominated, with facilitating habitat also influential in explaining gene flow. For subpopulations within Kittery West, results were variable among the different analytical methods and no clear landscape pattern emerged. This variability may have been a result of small sample sizes. Alternatively, at these small spatial scales, dispersal patterns may be more influenced by microsite characteristics or behavioral interactions between individuals.

Our clustering analyses suggested a barrier effect of the two interstates in our study area, I-95 and I-295, and in our MRM resistance modeling, the roads-as-barriers model explained the largest amount of genetic variation in the analysis across all populations. The size of the road is an important factor influencing dispersal, and it is likely that only major roads with high traffic loads are substantial barriers to dispersal (e.g., Frantz et al. 2010). Nevertheless, the logarithmic road model, which placed an extremely high dispersal cost on the interstate, was the only road model that was not significant in the Mantel tests across all populations, suggesting that even major highways are not absolute barriers. Further, the linear cost model, for which costs increase incrementally with road class, was the top road model for analyses across and within all populations. Accordingly, in support of an incomplete barrier effect of roads, we found genetic similarity of cottontails that occupied patches on either side of I-95 in Kittery, adjacent to the highway. Dispersal between these patches may have occurred through a culvert that passes underneath the highway in this location. Alternately, this connectivity might be a result of an occasional individual successfully crossing the highway, as has been observed by radiotelemetry (J. Litvaitis, pers. obs.; H. Holman, New Hampshire Fish and Game, pers. comm.). Underpasses with shrubby riparian habitat may also facilitate cottontail dispersal across interstates. Such an underpass occurs in the vicinity of the Portland Jetport and, in combination with historical occupancy of previously suitable habitat patches (discussed below), may explain the genetic connectivity observed between the Jetport individuals (east of I-95) and those in Kittery West (west of I-95), despite the barrier posed by the interstate. Large highways have been found to restrict movement in other small- to mid-sized mammals, such as badgers (*Meles meles;* Mata et al. 2008) and pygmy rabbits (*Brachylagus idahoensis;* Estes-Zumpf et al. 2010), although they may use permeable features, including culverts and underpasses (Mata et al. 2008).

Despite the widely recognized negative ecological effects of roads (Balkenhol and Waits 2009; Fahrig and Rytwinski 2009), they appear to have a complex effect on natural populations that may vary with focal species, population size, and road type (Clevenger et al. 2001; Forman et al. 2003; Gauffre et al. 2008). Roads, which are often associated with adjacent strips of herbaceous and shrubby vegetation, can create and enhance habitat for some species (Bissonette and Rosa 2009; Fahrig and Rytwinski 2009) and thereby serve as movement and dispersal corridors rather than barriers (Crooks and Sanjayan 2006). Indeed, roads may enhance gene flow for some generalist and invasive species (Crispo et al. 2011; Laurence et al. 2013). Species that specialize on early successional habitats, including the New England cottontail, however, may be faced with conflicting positive and negative effects of roads, which may facilitate dispersal through suitable road*side* habitat, while simultaneously increasing mortality risk through road crossings (Tash and Litvaitis 2007). Given these dual facilitating and barrier effects, interstate highways may have an effect similar to that of drift fences for cottontails, which may avoid the high volume roads and be more likely instead to travel along them, utilizing the adjacent shrubby habitat to avoid crossing them (e.g., Forman et al. 2003; Holderegger and Di Giulio 2010).

We were able to evaluate the roads-as-dispersal-facilitators hypothesis, in part, through our landscape resistance modeling, in which the facilitator model accounted for the facilitating effects of roadsides and other linear thicket conduits. The facilitator models were significant in most of the within-population analyses and explained a larger portion of the genetic variation than the barrier effects of roads, surficial water, or geographic distance within populations, except for Kittery East, which showed a pronounced pattern of isolation by distance. The potential facilitating effects of linear shrub-lined conduits were further illustrated by the Bayesian clustering results of genetic similarity of the Jetport and Kittery West populations, separated by a distance of 48 km. This genetic similarity likely reflects recent historical connectivity. A major powerline runs parallel to and on the west side of the interstate between these two populations, and cottontails occupied habitat patches within this intervening area as recently as 2000–2003 (Litvaitis et al. 2006; Fig. 2). The shrubby habitat along this powerline and along the interstate itself may have served as a north–south dispersal corridor, connecting these patches in the recent past. Although the habitat patches between Kittery West and the Jetport were either no longer suitable or unoccupied during our surveys, we found cottontails within and adjacent to the powerlines within Kittery West. While our results highlight the importance of linear conduits as dispersal facilitators, our approach did not allow us to fully evaluate the relative importance of the various facilitating features (roadsides, powerlines, and railroads), as we did not test different permutations of the relative costs for each feature. Our findings suggest that the relative importance of the facilitating features may depend on the landscape matrix composition, as the best-supported facilitator cost model (highest Mantel *r*) varied by population. Teasing apart these influences is a potentially important avenue for future research. Additionally, although our analyses focused on shrubland habitats, future studies should investigate the potential facilitating effects of other types of early successional habitats, such as tall grasslands and hayfields, and fully evaluate the role of other habitats and land-cover features in influencing cottontail dispersal.

Our landscape models and Bayesian clustering results also revealed the influence of the Salmon Falls/Piscataqua River as a partial barrier in the Kittery West population, separating individuals to its east and west. The Piscataqua River ranges 250–500 m wide where it empties into the Atlantic Ocean and is approximately 50 m wide to the northwest, where it becomes the Salmon Falls River. One of the two migrants identified by assignment tests was a putative disperser across the river (sampled on the east side in KW-ME and assigned to KW-NH on the west side), suggesting that cottontails do disperse, at least occasionally, across the river. Although rivers pose barriers to the dispersal of many small- and medium-sized mammals (e.g., Cullingham et al. 2009; Chambers and Garant 2010; Frantz et al. 2010), several species of rabbits, including other *Sylvilagus*, have been reported to swim (Chapman and Feldhamer 1981; Chapman and Willner 1981; Estes-Zumpf and Rachlow 2009). The permeability of different waterways may vary, however, according to their width, flow, winter ice cover, or surrounding landscape shape (Cullingham et al. 2009; Frantz et al. 2010). Small islands that occur in the narrower portion of this river may further facilitate occasional crossing by New England cottontails. These findings also bear relevance to understanding the distribution of nonnative eastern cottontails (*Sylvilagus floridanus*) in this region, which extended their range into southern New Hampshire in the late 1960s (Jackson 1973). Our results support the contention that the Piscataqua River has been a dispersal barrier preventing the spread of eastern cottontails from southern New Hampshire into Maine. Yet, these findings also raise concerns about the potential for eastern cottontails to cross the narrower portions of this river if they continue their spread farther northeast into New Hampshire.

## Conclusion

This study found negative genetic consequences of fragmentation and influences of landscape structure on gene flow for a habitat specialist. Our findings of isolated populations with low effective population sizes and low genetic diversity suggest that the New England cottontails in Maine and Seacoast New Hampshire are vulnerable to extirpation without immediate human intervention. Extensive habitat loss and fragmentation have reduced the availability of suitable thicket habitat in this region, such that fewer and smaller habitat patches exist, separated by increasingly large geographic distances. As a result, occupancy has declined, and remaining cottontails are effectively isolated into small populations, within which genetic drift occurs and genetic diversity is being lost in the absence of gene flow. Genetic data revealed historical connections among remnant populations, a finding that points toward the importance of restoring suitable habitat to reconnect these populations. Landscape resistance models also showed the importance of linear conduits of thicket habitats (powerlines, roadsides, railways) in sustaining gene flow and the role of major highways and waterways in impeding dispersal. We also found evidence that anthropogenic connections, such as underpasses and possibly culverts, may be effective in facilitating dispersal across interstate highways.

Management to create additional suitable habitat is critical for restoration of cottontail populations in this region. This habitat creation has been the dominant focus of a recent conservation initiative. The current goals of the conservation strategy for the New England Cottontail (Fuller and Tur 2012) outline targets for the size, number, and proximity of restored habitat patches per each designated focal management area. Given the critically low effective population sizes, however, habitat creation alone may be an insufficient management solution and translocations may be necessary to augment existing populations. In addition, the creation of dispersal corridors, such as expanding roadside shrubby edge and potentially mitigating highway crossings via underpasses or culvert modifications (e.g., Dodd et al. 2004), may also be effective in restoring connectivity in this highly fragmented landscape. Our findings in this study highlight the need for considering not only the number of hectares restored, but also the placement and configuration of habitat patches to afford gene flow within restoration landscapes. Our results provide a starting point for addressing the broader goal of designing conservation landscapes that support viable, functionally connected metapopulations with the potential to persist in the long term. Doing so will require establishing functional connections both within and among focal management areas.

## Data Accessibility

Microsatellite genotypes and sampling locations are available in Dryad (doi:10.5061/dryad.1s834) following a 2-year embargo period.

Genotyping protocols and cost values used in landscape resistance modeling are provided in manuscript tables; additional information on the derivation of GIS layers for landscape modeling is provided in the Appendix 1.

## Appendix 1: GIS Data Sources and General Processing Methods

### Roads Model

#### Data source

- U.S. Geological Survey (USGS), Maine Office of Geographic Information Systems (MEGIS) (ed.) *trans, 1989: Augusta, Maine*. Available online at http://megis.maine.gov/catalog. Accessed 3 May 2010.

*We obtained the “trans” feature dataset from Maine GIS* (*which extends into the small area of New Hampshire included in our dataset*) *and clipped it to a rectangle outlining the extent of our sampling locations. This file includes roads, trails, pipelines, railroads, and powerline utility corridors* (*“Otrans”*) *to be used in the development of the facilitator models. To create a vector file of the roads, we first selected the class 1–6 roads and buffered the polyline roads by class, such that busier roads had a wider buffer, which would translate into wider polygons for interstates versus trails, for example. Buffer sizes were as follows: roads classified as “interstate” were buffered to 30 m, primary roads 20 m, secondary roads 10 m, improved roads 5 m, unimproved roads 2 m, and trails 1 m*.

### Surficial Water Model

#### Data source

- U.S. Geological Survey (USGS) and U.S. Environmental Protection Agency (USEPA)*. National Hydrography Dataset* (*NHD*) *Medium Resolution*, 2005; updated in 2006 with SOSC (Augmenting NHDPlus Strahler order values using Strahler calculator): Corvallis, Oregon. Available online at http://nhd.usgs.gov. Accessed 25 March 2011.

*To create a model of surficial water, we first merged the NHDwaterbody* (*lakes, ponds, etc*.) *and NHDArea* (*rivers, inlets, etc*.) *files. This output was then joined with the NHDflowline shapefile, based on spatial location, such that the maximum Strahler stream order class was assigned to each water feature. Water features without stream order information were given a stream order of 1*.

### Facilitator Model

#### Data sources

- U.S. Geological Survey (USGS), Maine Office of Geographic Information Systems (MEGIS) (ed.) *trans, 1989: Augusta, Maine*. Available online at http://megis.maine.gov/catalog. Accessed 3 May 2010.
- *Roads*. Created by Lindsey Fenderson, as a modification of *trans*, using ArcGIS 9.3, as described above. (May 2010).

*Road edges were established by buffering class 1–2 roads from the above-created “Roads” file by 30 meters and buffering class 3–6 roads by 10 m* (*as verges maintained on highways are typically wider than those found on reduced-volume roads*)*, then erasing the roads themselves. Powerline and railroad features were obtained from the “Otrans” of the clipped “trans” dataset. These polylines were buffered by 30 m and combined with the “RdEdges” shapefile for the final “Facilitators” dataset*.

*All vector models* (*roads, surficial water, and facilitators*) *were then converted to rasters with a cell size of 10 for cost-distance analyses. Raster files were reclassed according to values given in Table* 2 *to develop each cost model per feature type* (*binary, linear, exponential, and logarithmic*).
